# Exploring Quantitative Biological Major, Trace, and Ultratrace Elements Composition and Qualitative Primary-Secondary Metabolites in *Lamiaceae* Medicinal Plants from Turkey

**DOI:** 10.1007/s12011-024-04219-z

**Published:** 2024-05-14

**Authors:** Enes Tekman, Tugay Asgarlı, Hafize Yuca, Alptuğ Atila, Ömer Çeçen, Songül Karakaya

**Affiliations:** 1 Department of Pharmaceutical Botany, Faculty of Pharmacy, Ataturk University, 25240 Erzurum, Turkey; 2 Department of Pharmacognosy, Faculty of Pharmacy, Ataturk University, 25240 Erzurum, Turkey; 3 Department of Analytical Chemistry, Faculty of Pharmacy, Ataturk University, 25240 Erzurum, Turkey; 4 Department of Plant and Animal Production, Vocational School of Technical Sciences, Karamanoğlu Mehmetbey University, 70200 Karaman, Turkey

**Keywords:** Elemental analysis, ICP-MS, *Lamiaceae*, Medicinal plants, Metabolites

## Abstract

Medicinal plants comprise a spectrum of constituents, encompassing both organic and inorganic elements. Elemental composition of 27 species of medicinal plants of *Lamiaceae* (including 17 endemic) family grown in Turkey was carried out by ICP-MS. The following elements were determined in analysed samples: Na, Mg, Al, K, Ca, Sc, Cr, Mn, Fe, Co, Zn, As, Rb, Sr, Cs, Ba, La, Ce, Sm, U, Se. Quantitative analysis of specific primary and secondary metabolites was carried out. Na and K are major constituents in plants. The concentrations of Na range from 332,495.590 g/kg (in sample 10SA) to 279,690.674 g/kg (in sample 4SA), while those of K vary from 67,492.456 g/kg (in sample 15SA) to 3347.612 g/kg (in sample 1A). Some metals such as Al, Cr, Mn, Fe, Co, Zn, As, Se, Rb, Sr, Cs, and Ba were also detected. Flavonoids, carbohydrates and tannins were present in all sample. Saponins were found in all samples except 1C and 2O. Coumarin were detected in samples 2N, 1 T, 1O, 1Z, 3SA, 1C, 4SA, 6SA, 8SA, 1 M, 11SA, 13SA, 2O, 14SA, 1H, and 16SI. Lipids were present in samples 6S, 9S, 1A, 10S, 1 M, 11SA, 12SA, 13SA, 14SA, and 16SI. Plants contain essential, rare earth, and trace elements at mg/kg concentrations, while major elements such as K and Na are present in high levels. Toxic element As (arsenic) was detected in all analyzed plants, but in most samples, its concentration was below the threshold set by World Health Organization.

**Graphical Abstract:**

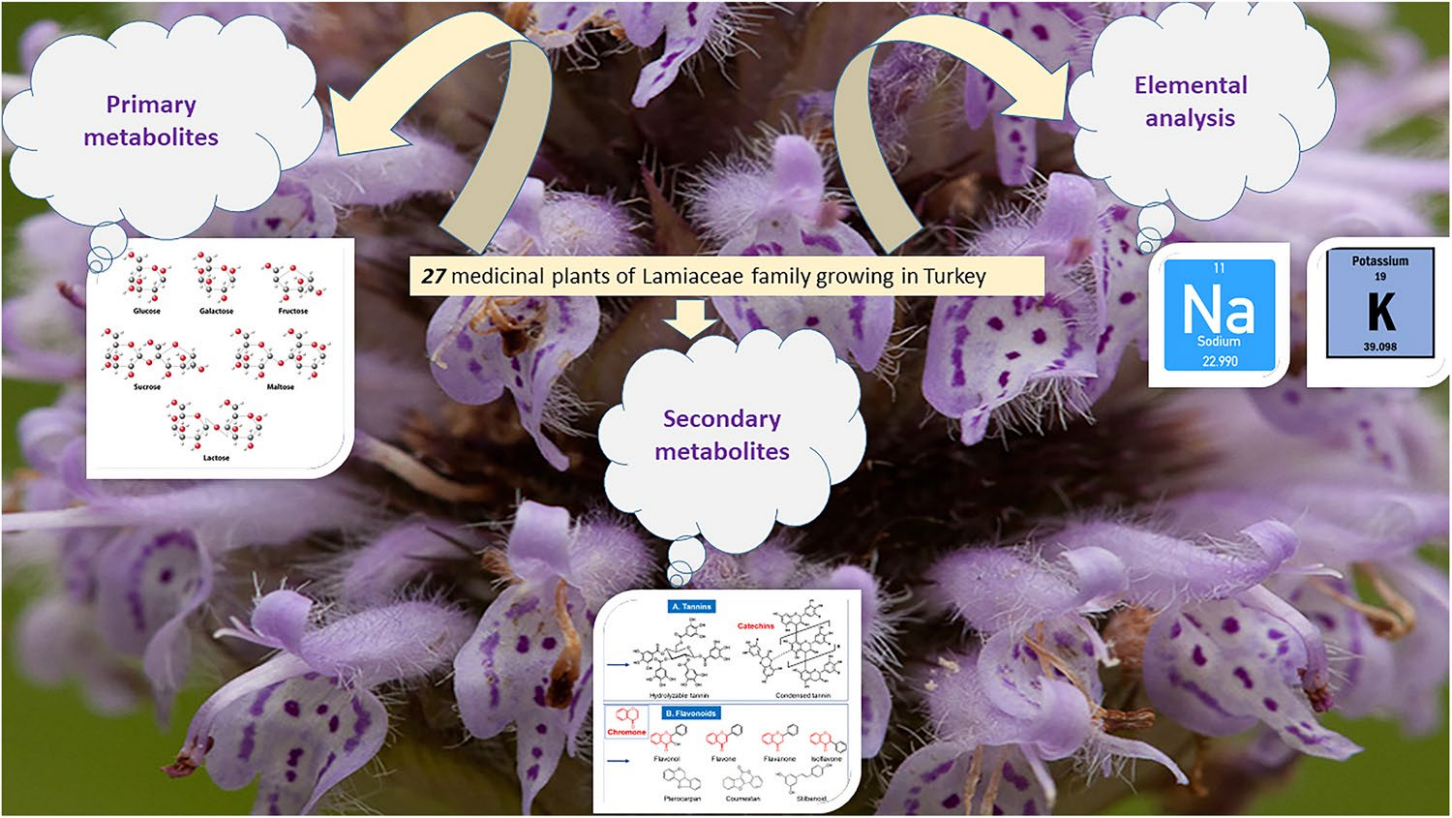

## Introduction

Medicinal plants comprise a spectrum of constituents, encompassing both organic and inorganic elements. The presence of macronutrients and trace (micro-) elements in medicinal plants serves as a rich source, playing a pivotal role in preventing a myriad of diseases [1]. Herbal remedies derived from medicinal plants have been employed as therapeutic agents since ancient times. Various plant components, including leaves, flowers, stems, roots, seeds, and bark, are utilized either individually or in synergistic combinations for their medicinal properties. The efficacy of these plants lies in their bioactive phytochemical constituents, which elicit specific physiological responses in the human body. Phytochemicals can be broadly categorized into two groups based on their functions within the plant. Primary metabolites (PMs), such as sugars, amino acids, proteins, lipids, and chlorophyll, are essential for basic growth processes. In contrast, secondary metabolites (SMs), including alkaloids, essential oils, flavonoids, tannins, terpenoids, saponins, phenolic compounds, and cardiac glycosides, play a crucial role in the plant's defense mechanisms against herbivores and other inter-species threats. These SMs contribute to the intricate pharmacological profile of medicinal plants, making them valuable resources in traditional and modern medicine alike. SMs in plants serve not only as a diverse array of natural products but also play a crucial role in fortifying the plant's defense mechanisms against pathogenic assaults and environmental stressors. Possessing noteworthy biological activities, these plant SM are gaining prominence as key components in medicinal formulations and food additives, catering to therapeutic, aromatic, and culinary needs. Research into plant secondary metabolites has witnessed a steady rise over the past five decades [2–5].

In the realm of agricultural science, prevailing challenges affecting global crop production encompass issues related to nutrient management, the presence of heavy and toxic metals, and the quest for optimal plant productivity. Plants, enduring for numerous decades, exhibit a diverse composition of elements in varying proportions. The elements present in plants are broadly categorized as either macronutrients, including Ca, K, Mg, N, P, and S, or micronutrients such as B, Cu, Cl, Fe, Mn, Mo, Na, and Zn. These elements play a pivotal role in the developmental processes and growth of plants, underscoring their significance in the agricultural landscape [6]. Plants frequently assimilate nutrients in quantities surpassing their immediate requirements, necessitating the storage of these excess nutrients within plant tissues until they are needed. Despite the selective uptake of essential micro- and macronutrients and the implementation of sophisticated exclusion mechanisms, plants unavoidably absorb elements that possess toxicity [7]. Elements exist in various forms in nature, and their presence is indispensable for the body to execute diverse functions. Trace elements hold paramount importance in facilitating cellular functions at biological, chemical, and molecular levels. These elements play a pivotal role in orchestrating important biochemical reactions by serving as cofactors for numerous enzymes and acting as central components for stabilizing the structures of enzymes and proteins. Certain elements exert control over crucial biological processes by binding to molecules on the receptor sites of cell membranes or by altering the membrane structure to impede the entry of specific molecules into the cell. The functions of trace elements exhibit a dual nature; at normal levels, they are crucial for stabilizing cellular structures, yet in deficiency states, they may stimulate alternative pathways, leading to various diseases. Disruptions in the balance of trace elements can lead to the onset of pathological conditions and diseases [8, 9].

The *Lamiaceae*, commonly known as the mint family, exhibits a broad distribution across various natural ecosystems, encompassing 236 genera. Characterized by square stems in cross-section, opposite leaves, and zygomorphic flowers with five united petals and sepals, these aromatic plants are cultivated for their straightforward propagation through methods such as stem cutting [10]. The *Lamiaceae* (Labiatae) family boasts numerous medicinal plants of considerable value. Within this family, there are an estimated 6900 to 7200 species. Among the most prolific genera are *Salvia* (with approximately 900 species), *Scutellaria* (360 species), *Stachys* (300 species), *Plectranthus* (300 species), *Hyptis* (280 species), *Teucrium* (250 species), *Vitex* (250 species), *Thymus* (220 species), and *Nepeta* (200 species) [11]. *Lamiaceae* stands as the third-largest family in terms of taxon number and the fourth-largest based on species count in Turkey. With 48 genera and a total of 782 taxa (603 species, 179 subspecies, and varieties), 346 of these taxa (comprising 271 species, 75 subspecies, and varieties) are endemic, constituting approximately 44% of the family's diversity in the country (data updated as of February 1, 2017). Additionally, there are 23 hybrid species, 19 of which exhibit endemism (82%). These findings underscore Turkey's significance as one of the primary centers of diversity for *Lamiaceae* in the Old World. The flora of Turkey holds significant importance for the *Lamiaceae* family [10, 12]. *Lamiaceae* is celebrated for housing a variety of active secondary metabolites that hold substantial biological and economic significance. These compounds encompass volatile oils containing monoterpenes and sesquiterpenes, diterpenes, triterpenes, phenolic acids, flavonoids, and other substances, each contributing diverse properties [13].

The majority of species within this plant family are known for their aromatic qualities and contain essential oils, rendering them highly valuable in the fields of cosmetics, perfumery, agriculture, food, and medicine [14]. Throughout history, the species belonging to the *Lamiaceae* family have held a storied tradition of utilization for flavoring, food preservation, and medicinal applications, owing to their dual benefits of healing and preventative attributes. It is widely acknowledged that each species possesses a unique and intricate blend of bioactive compounds, where each component plays a role in its overall bioactivity. These plants are highly esteemed for their capability to produce an extensive array of secondary metabolites, showcasing potent antibacterial, antioxidant, anti-inflammatory, antimicrobial, antiviral, and anticancer activities [15].

The trace elements are presently recognized and classified by the World Health Organization (WHO) into three groups: essential elements, elements considered likely to be essential, and elements with potential toxicity. Man necessitates essential trace elements in quantities spanning from 50 µg to 18 mg per day, wherein they function as catalytic or structural components within larger molecules [16]. In our study, the major elements identified in *Lamiaceae* plants are sodium (Na) and potassium (K). Sodium and potassium play indispensable roles in human health, serving as crucial ions within the body and being intricately linked to numerous physiological and pathophysiological processes [17].

In this research, we undertook an evaluation of various elements in 27 (including 17 endemic) herb species belonging to the *Lamiaceae* family, gathered in Turkey, utilizing ICP-MS analysis. Moreover, a quantitative analysis of specific primary and secondary metabolites was carried out. A substantial number of the analyzed herbs are readily available and widely utilized voluntarily by the entire population of the country.

## Material and Method

### Plant Materials

**Fig. 1:**
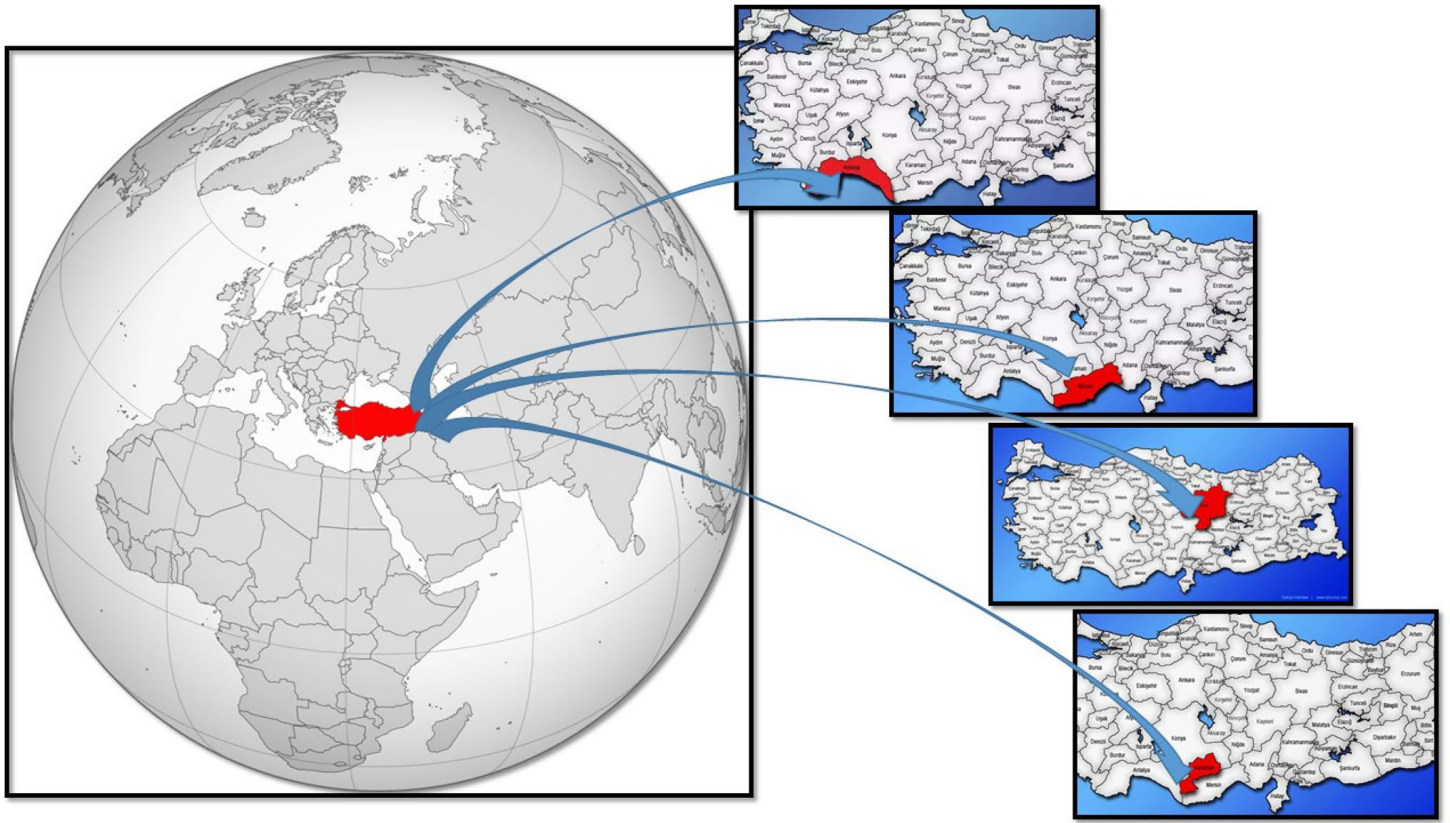
A map showcasing the plant collecting sites across Turkey

The names of the plants used, the localities where they were collected, their herbarium numbers and endemism status are given in Table 1. A map showcasing the plant collecting sites across Turkey is presented in Fig. 1.

**Table 1:** The names of the plants used, the localities, collection dates where they were collected, their herbarium numbers and endemism status

Species/Taxon	Local Names	Locality-Collection Dates	Herbarium number	Endemism
*Nepeta cilicica* Boiss	Gök Pisikotu	Mersin Province, Anamur District, Abanoz Village vicinity, 1650 m, 16 June 2021	KMUB 7005	Endemic
*Nepeta isaurica* Boiss. & Heldr	Kırk Pisikotu	Mersin Province, Anamur District, Sugözü Village, Tamtır Plateau, 1952 m, 16 June 2021	KMUB 6995	Endemic
*Thymus cilicicus* Boiss. & Balansa	Kekik Limonkekiği, Kılıçkkekiği, Peynir Kekiği, Kılçıkkekiği	Karaman Province, Bucakkışla Village vicinity, 450 m, 13 June 2022	KMUB 7380	
*Origanum leptocladum* Boiss	Bayırmercanı	Karaman Province, Ermenek District, Eskice Village vicinity 850 m, 11 July 2022	KMUB 7364	Endemic
*Ziziphora clinopodioides* Lam	Dağreyhanı Reyhan	Karaman Province, 6 km from Ermenek District to Balkusan Village 1443 m, 11 July 2022	KMUB 7366	
*Sideritis hololeuca* Boiss. & Heldr	Çalıçayı Dağ Çayı	Between Karaman Province Mut District, Alaoda church surroundings, 1200 m, 7 July 2021	KMUB 4969	Endemic
*Salvia tomentosa* Mill	şalba Adaçayı, salba, hoşaflama, moşafla, moşapla	Karaman Province, Ermenek District, Gökçekent Village vicinity, 854 m, 25 June 2019	KMUB 5322	
*Salvia pisidica* Boiss. & Heldr. ex Benth.	Eldiven Çayı, Çay, Sarı Çay, Hava Otu	Antalya Province, Elmalı District surroundings 1245 m, 21 May 2021	KMUB 6962	Endemic
*Cyclotrichum origanifolium* (Labil) Manden&Schenk	Dağnanesi Mentol, Mentolnane, Tüter Ot	Karaman Province, Kazımkarabekir District, Akarköy Village Masdatbeli region, 1234 m,	KMUB 5517	
*Salvia heldreichiana* Boiss. ex Benth.	Ayaklı Şalba	Karaman Province, Lale Village surroundings, 1256 m, 16 July 2020	KMUB 6269	Endemic
*Salvia absconditiflora* (Monlbret & Aucher ex Benth.) Greuter & Burdet	Karaşalba Boz Şabla, Kara Şabla, Karaot, Yabani Adaçayı	Mersin Province, Mut District, Sertavul Gateway, Akçeşme vicinty, 1345 m, 21 May 2019	KMUB 5104	Endemic
*Salvia quezelii* Hedge & Afzal-Rafii	Limon Adaçayı	Mersin Province, Anamur District, Boğuntu Village vicinty, 700 m, 09 May 2020	KMUB 6000	Endemic
*Sideritis libanotica* Labill. subsp. *violascens* (P.H.Davis) P.H.Davis	Topukluçay; Dağ Çayı	Karaman Province Ermenek District, Tekeçatı Plateaeu, 1100 m, 16 July 2021	KMUB 7157	Endemic
*Nepeta sulfuriflora* P.H.Davis	Sarı Pisik Otu	Mersin Province, Anamur District, Boğuntu Village vicinty, 750 m, 16 June 2021	KMUB 6329	Endemic
*Salvia caespitosa* Montbret & Aucher ex Benth	Kırk Şalba	Karaman Province Ermenek District, Pancar Fountain vicinity, 1134 m, 21 July 2021	KMUB 7134	Endemic
*Salvia dichroantha* Stapf	Kutnu Yağlıkara	Karaman Province, Sudurağı Village vicinity, 1025 m, 25 June 2020	KMUB 5498	Endemic
*Ajuga chamaepitys* (L.) Schreb. subsp. *chia* (Schreb.) Arcang	Acıgıcı Bodur Ot, Bozca Ot, Kokar Ot, Mayasıl Otu, Yer Çamı, Yermeşesi	Karaman Province, Karamanoğlu Mehmetbey University Campus, 1034 m, 22 May 2022	KMUB 7321	
*Salvia viridis* L	Zarifşalba Adaçayı, Yeşilbaş	Mersin Province, Tarsus District, Şamlar Village, Canyon of Keşbükü, 150 m, 27 April 2021	KMUB 6875	
*Marrubium globosum* Montbret et Aucher subsp.* globosum*	Bozcaboğum Amel Otu, Beyaz Şabla, Boz Ot	Karaman Province, Kazımkarabekir District vicinity, 1085 m, 13 June 2022	KMUB 7379	Endemic
*Salvia aucheri* Benth. subsp. *canescens* (Boiss. & Heldr.) Celep, Kahraman & Doğan	Koramaz; Dağ Çayı	Karaman Province, Lale Village surroundings, 1256 m, 13 June 2022	KMUB 7377	Endemic
*Salvia blepharochlaena* Hedge& Huber- Morath	Hoş Şalba	Karaman Province Vicinity, 1095 m, 11 July 2020	KMUB 5017	Endemic
*Salvia candidissima* Vahl. subsp. *occidentalis* Hedge	Ak Galabor	Karaman Province, Ermenek District, Kazancı village, Beşkuyu surroundings, 950 m, 20 June 2020	KMUB 5452	
*Origanum vulgare* L	Karakınık; Arigani, Kekik, Merdus	Karaman Province, Ermenek District, Gökçeseki village, Bağarası region, 1068 m, 12 June 2022	KMUB 7365	
*Salvia albimaculata* Hedge & Hub.-Mor.	Hoşdudak	Karaman Province, Ermenek District Vicinity, 1287 m, 24 May 2021	KMUB 6928	Endemic
*Salvia hypargeia* Fisch. C. A. Mey	Siyahot Kök Çayı	Karaman Province, Ermenek District, Balkusan village vicinity, 1248 m, 13 July 2021	KMUB 7378	Endemic
*Hyssopus officinalis* L. subsp.* officinalis*	Zufaotu Çördük	Sivas Province, Zara District, Geminbeli Gateway, 1654 m, 03 August 2021	KMUB 7271	
*Sideritis argyrea* P.H. Davis	Bozeşekçayı	Mersin Province Anamur District Sugözü Village Tamtır Plateaeu, 1789 m, 13 August 2021	KMUB 5371	Endemic

*Abbreviations*: 1N, *Nepeta cilicia*; 2N, *Nepeta isaurica*; 1 T, *Thymus cilicicus*; 1O, *Origanum leptocladum*; 1Z, *Ziziphora clinopodioides*; 1SI, *Sideritis hololeuca*; 2SA, *Salvia tomentosa*; 3SA, *Salvia pisidica*; 1C, *Cyclotrichium origanifolium*; 4SA, *Salvia heldreichiana*; 5SA, *Salvia absconditiflora*; 6SA, *Salvia quezelii*; 7SI, *Sideritis libanotica* subsp. *violascens*; 3N, *Nepeta sulphuriflora*; 8SA, *Salvia caespitosa*; 9SA, *Salvia dichroantha*; 1A, *Ajuga chamaepitys* subsp. *chia*; 10SA, *Salvia viridis*; 1 M, *Marrubium globosum* subsp. *globosum*; 11SA, *Salvia aucheri* subsp. *canescens*; 12SA, *Salvia blepharochlaena*; 13SA, *Salvia candidissima* subsp. *occidentalis*; 2O, *Origanum vulgare*; 14SA, *Salvia albimaculata*; 15SA, *Salvia hypargeia*; 1H, *Hyssopus officinalis* subsp. *officinalis*; 16SI, *Sideritis argyrea*

### Extraction

The extraction process utilized in the study involved a mobile maceration technique, wherein 10 g of the dried flowering above-ground parts of each plant were meticulously weighed and then transformed into powder form. Subsequently, this powdered plant material was subjected to a mobile maceration process with 150 ml methanol (after 8 h, it was filtered and 150 ml of methanol was added again and this process was continued for 3 days), which typically involves soaking the plant material in a solvent at room temperature for a specific duration. During the mobile maceration process, the powdered plant material was immersed in a suitable solvent. The choice of solvent can vary depending on the specific compounds being targeted for extraction and their solubility characteristics. Commonly used solvents for maceration with methanol. The duration of maceration typically ranged from 3 to 8 h at room temperature. This duration allows sufficient time for the solvent to penetrate the plant material and extract the desired compounds. After the designated maceration period, the filtrates obtained from each day's extraction were systematically combined. Subsequently, the combined filtrates underwent evaporation using a rotavapor. Rotavapor, short for rotary evaporator, is a common laboratory instrument used for gentle evaporation of solvents from a mixture under reduced pressure. This process helps concentrate the extracted compounds, leaving behind a more concentrated extract for further analysis. Overall, the mobile maceration process employed in the study involved soaking the powdered plant material in a solvent for a specific duration at room temperature to facilitate the extraction of bioactive compounds. This method allows for efficient extraction while preserving the integrity of the extracted compounds. At the conclusion of each day, the filtrates were systematically amalgamated and subjected to evaporation using a rotavapor.

## Qualitative Analysis of Secondary and Primary Metabolites

### Detection of Alkaloids

The methanolic extract of each species was weighed 0.5 g, 10 ml of 70% ethanol solution (containing 6% H_2_SO_4_) was added, boiled for 1 min and was cooled. Some of the extract was separated into 2 tubes and Mayer and Dragendorff reagents were added. The samples were checked for the appearance of precipitates and further experiments were continued for the samples in which precipitates were observed. After this control, the ethanol extract was alkalinized with a 25% Na_2_CO_3_ solution. Then, extracted with 15 ml of chloroform, the chloroformed layer was taken into a clean separating funnel and extracted with 15 ml of 10% acetic acid solution. The acetic acid phase was divided into three separate tubes and one tube was kept as a control, the second tube added Mayer's reagents and the third tube added Dragendorff's reagents. It was checked for the precipitate formation [18–20].

### Detection of Flavonoids

The methanolic extract of each species was weighed 0.5 g and extracted with water. The test solution was extracted with ethyl acetate into a separatory funnel and the ethyl acetate phase was capsulated and dried in a water bath. The residue remaining in the capsule was dissolved with a mixture of HCl:MeOH:H_2_O (1:1:1) and transferred to a test tube. After adding Mg powder, the color was observed and the flavonoid was identified [18–20].

### Detection of Cardioactive Heterosides

The methanolic extract of each species was weighed 0.5 g and prepared as a solution with 10 ml 70% ethanol. Add 1 ml of concentrated lead subacetate solution to the solution, mixed and filtered. The filtrate was extracted with 10 ml of chloroform, the chloroform layer was separated, taken into a capsule and evaporated. Add 3 ml of 3.5% glacial acetic acid solution of FeCl_3_ to the capsule and carefully transfer to 2 ml of concentrated sulfuric acid in a test tube to form a layer. The colors seen on the surface of the separation and in the acetic acid layers were observed [18–20].

### Detection of Saponosides

The methanolic extract of each species was taken 0.5 g and placed in a test tube with 10 ml of hot water. After cooling, the tube was shaken strongly for 10 s. If saponoside was present, a 1–10 cm high foam layer was observed which remained stable for at least 10 min. Then 1–2 drops of 2N HCl were added and the persistence of the foam layer was observed. The methanolic extract of 0.5 g of each species was dissolved in chloroform and filtered. Add an equal volume of concentrated H_2_SO_4_ to the filtrate. The presence of fluorescence color in the chloroform layer and in the acidic layer was observed. The reaction is based on the coloration of the [18–20].

### Detection of Anthocyanosides

The methanol extract of each species was extracted in 50% ethanol. The extract was filtered, the filtrate was separated into five portions and the following reactions were performed:

- The addition of diluted H_2_SO_4_ resulted in the formation of red color.
- First NaOH solution was added, then diluted HCl was added and the colors were observed.
- The precipitate was observed with a 10% lead acetate solution.
- Added a volume of amyl alcohol, shaken and observed the coloration of the layers [18–20].

### Detection of Tannins

The methanol extract of each species was taken in a spatula to make an aqueous extract. The extract was divided into 3 parts and the following reactions were applied:

- 2 ml of 1% 1% saline gelatin solution (saturated with NaCl) was added and the precipitate was observed.
- Tannins give a color reaction with iron salts. This reaction was determined with 5% FeCl_3_. Olive green and blue-black color formation was observed.
- Brominated water was added and the precipitate were observed [18–20].

### Detection of Anthracene Heterosides

The methanol extract of each species was taken at 0.5 g and boiled with diluted H_2_SO_4_. After filtering while hot, the filtrate was cooled. The extract was extracted with ether to eliminate the ether layer and shaken with 10% ammonia solution. The color in the underlying ammonia layer was observed [18–20].

### Detection of Coumarin

The methanol extract of each species was taken 0.5 g, extracted in 10 ml ethanol and filtered. The filtrate was concentrated to dryness in a capsule and 1N NaOH solution was added. It was transferred into a tube and checked for fluorescence color at UV 366 nm [18–20].

### Detection of Starch

The methanol extract of each species was taken 0.5 g and aqueous extract was prepared. Add a few drops of 0.1 N Iodine solution. The formation of purple color was checked [18–20].

### Detection of Lipid

The extract of each species was made with petroleum ether and 5 ml was taken in a glass tube. It was concentrated in a water bath and applied as a stain on a filter paper. The filter paper was allowed to dry in an oven at 100 °C for 5 min. The oily stain was observed on the paper [18–20].

### Detection of Carbohydrates

The methanol extract of each species was weighed 1 g and 6 ml of water was added to make an aqueous extract. It was filtered and the filtrate was divided into 3 tubes. The following experiments were applied to the solutions in the tubes respectively:

- 2 ml of Fehling A and 2 ml of Fehling B solutions were added to an empty tube and mixed. Then the test solution was added to it. The tube was heated and red colored precipitate.
- 1–2 drops of 15% solution of 1-naphthol in ethanol was added to 1 ml of the test solution. To this mixture, 1 ml of concentrated H_2_SO_4_. Purple ring formation was observed between the two layers.
- Add a few crystals of resorcin to 1 ml of test solution. To this 1 ml of concentrated HCl was added and kept in a water bath. The presence of red color was observed [18–20].

### Detection of Cyanogenetic Heterosites

0.5 g of the methanol extract of each species was taken into a flask and some water was added. The filter paper was wetted first with picric acid and then with sodium carbonate solution. The wetted filter paper was dropped into the flask. The flask was heated and red color formation was observed on the yellow colored filter paper [18–20].

### ICP-MS Analysis of 21 Elements

The determination of elemental concentrations in the solution was carried out using an Inductively Coupled Plasma-Mass Spectrometer (ICP-MS), specifically the Agilent 7800 series provided by Agilent Technologies in Japan. For sample introduction, a glass MicroMist nebulizer from U-series in Australia and a double-pass quartz spray chamber from the USA were employed. The plasma system included a quartz torch (2.5 mm) and nickel components, including a sample cone and skimmer cone for the x-lens. Before sample analysis, all quartz and nickel components underwent a meticulous cleaning process. Quartz and glass elements were soaked in a 5–10% HNO_3_ solution overnight, thoroughly rinsed with distilled water, dried in an oven, and then installed on the device. Nickel components, such as sample and skimmer cone pieces, underwent ultrasonic baths in pure water, 5% HNO_3_ solution, and distilled water successively for five minutes each. Following this, they were cleaned with a cotton ball, rinsed thoroughly with distilled water, and dried in the oven before installation on the device. Before analysis, the device underwent a 45-min helium gas purge. Activation was followed by adjusting parameters, including Plasma gas (15 L/min), auxiliary gas (1 L/min), carrier gas (1 L/min), makeup/dilution gas (1 L/min), and carrier gas pressure (1.45 kPa). The plasma gas, auxiliary gas, carrier gas, and makeup/dilution gas were Argon (Ar).Torch axis, resolution axis, EM, standard lenses tune, plasma correction, full spectrum, and performance report tests were carried out sequentially. Calibration of the device utilized a tuning solution (1 µg/L Ce, Co, Li, Mg, Tl, Y). The values obtained during tuning were scrutinized to identify any deviations in the device. Standard solutions, prepared from stock solutions, were then analyzed, and calibration curves were verified within the standard reference range (0–10, 25, 50, 100, 250, 500 g/kg, mg/kg, and µg/kg for Na, Mg, Al, K, Ca, Sc, Cr, Mn, Fe, Co, Zn, As, Se, Rb, Sr, Cs, Ba, La, Ce, Sm, and U elements.

## Results

### Qualitative Analysis of Secondary Metabolites

The qualitative analysis of primary and secondary metabolites for the samples is detailed in Table 2. Flavonoids were identified in all samples, while tannins were present in every sample. Saponins were found in all samples except 1C and 2O. Coumarin was detected in various samples, including 2N, 1 T, 1O, 1Z, 3SA, 1C, 4SA, 6SA, 8SA, 1 M, 11SA, 13SA, 2O, 14SA, 1H, and 16SI. Cardioactive heterosides were absent in all samples. Starch was identified solely in samples 4SA and 10SA. Lipids were present in samples 6S, 9S, 1A, 10S, 1 M, 11SA, 12SA, 13SA, 14SA, and 16SI, while carbohydrates were observed in all samples. Notably, no alkaloids, anthocyanosides, anthracene heterosides, or cyanogenetic heterosides were detected among the samples. The qualitative analysis data of primary and secondary metabolites for the methanolic extracts were provided in Table 2.

**Table 2:** Data of qualitative analysis of secondary and primary metabolites of methanolic extracts

Codes of plants	Alkoloid	Flavonoids	Anthocyanoside	Tannin	Saponoside	Coumarin	Anthracene Heterosides	Cardioactive Heterosides	Starch	Cyanogenetic Heteroside	Lipid	Carbohydrates
1N	-	+	-	+	+	-	-	-	-	-	-	+
2N	-	+	-	+	+	+	-	-	-	-	-	+
1 T	-	+	-	+	+	+	-	-	-	-	-	+
1O	-	+	-	+	+	+	-	-	-	-	-	+
1Z	-	+	-	+	+	+	-	-	-	-	-	+
1SI	-	+	-	+	+	-	-	-	-	-	-	+
2SA	-	+	-	+	+	-	-	-	-	-	-	+
3SA	-	+	-	+	+	+	-	-	-	-	-	+
1C	-	+	-	+	-	+	-	-	-	-	-	+
4SA	-	+	-	+	+	+	-	-	+	-	-	+
5SA	-	+	-	+	+	-	-	-	-	-	-	+
6SA	-	+	-	+	+	+	-	-	-	-	+	+
7SI	-	+	-	+	+	-	-	-	-	-	-	+
3N	-	+	-	+	+	-	-	-	-	-	-	+
8SA	-	+	-	+	+	+	-	-	-	-	-	+
9SA	-	+	-	+	+	-	-	-	-	-	+	+
1A	-	+	-	+	+	-	-	-	-	-	+	+
10SA	-	+	-	+	+	-	-	-	+	-	+	+
1 M	-	+	-	+	+	+	-	-	-	-	+	+
11SA	-	+	-	+	+	+	-	-	-	-	+	+
12SA	-	+	-	+	+	-	-	-	-	-	+	+
13SA	-	+	-	+	+	+	-	-	-	-	+	+
2O	-	+	-	+	-	+	-	-	-	-	-	+
14SA	-	+	-	+	+	+	-	-	-	-	+	+
15SA	-	+	-	+	+	-	-	-	-	-	-	+
1H	-	-	-	+	-	+	-	-	-	-	-	+
16SI	-	+	-	+	+	+	-	-	-	-	+	+

+ present, − absent

*Abbreviations*: 1N, *Nepeta cilicia*; 2N, *Nepeta isaurica*; 1 T, *Thymus cilicicus*; 1O, *Origanum leptocladum*; 1Z, *Ziziphora clinopodioides*; 1SI, *Sideritis hololeuca*; 2SA, *Salvia tomentosa*; 3SA, *Salvia pisidica*; 1C, *Cyclotrichium origanifolium*; 4SA, *Salvia heldreichiana*; 5SA, *Salvia absconditiflora*; 6SA, *Salvia quezelii*; 7SI, *Sideritis libanotica* subsp. *violascens*; 3N, *Nepeta sulphuriflora*; 8SA, *Salvia caespitosa*; 9SA, *Salvia dichroantha*; 1A, *Ajuga chamaepitys* subsp. *chia*; 10SA, *Salvia viridis*; 1 M, *Marrubium globosum* subsp. *globosum*; 11SA, *Salvia aucheri* subsp. *canescens*; 12SA, *Salvia blepharochlaena*; 13SA, *Salvia candidissima* subsp. *occidentalis*; 2O, *Origanum vulgare*; 14SA, *Salvia albimaculata*; 15SA, *Salvia hypargeia*; 1H, *Hyssopus officinalis* subsp. *officinalis*; 16SI, *Sideritis argyrea*

### Elemental Analysis

The analysis of the elemental composition of methanol extracts revealed notable variations in element concentrations, as outlined in Table 3. Using ICP-MS, the methanol extracts were investigated for the presence of 21 elements, including Na, Mg, Al, K, Ca, Sc, Cr, Mn, Fe, Co, Zn, As, Rb, Sr, Cs, Ba, La, Ce, Sm, U, and Se. The results indicated elevated levels of Na [ranging from 332,495.590 g/kg (in sample 10SA) to 279,690.674 g/kg (in sample 4SA) g/kg] and K [ranging from 67,492.456 g/kg (in sample 15SA) to 3347.612 g/kg (in sample 1A)]. Some heavy metals such as Al, Cr, Mn, Fe, Co, Zn, As, Se, Rb, Sr, Cs, Ba, La, Ce, Sm (except samples 1SI, 2SA-12SA), and U (including samples 15SA and 16 SI) were also detected.

**Table 3:** The Composition of Elemental Analysis of 27 *Lamiaceae *spp*.* Methanolic Extracts

Species	Major elements at g/kg Concentrations		Trace elements at mg/kg Concentrations													Ultratrace elements at µg/kg Concentrations					
	Na	K	Mg	Al	Ca	Sc	Cr	Mn	Fe	Co	Zn	Ba	Sr	Rb	Se	As	La	Ce	Cs	Sm	U
1SI	301,994.945	18,278.942	4098.195	8169.976	2959.973	102.682	299.319	50.979	6747.404	223.824	3269.026	83.601	18.444	6.421	15.846	6.760	0.488	1.341	0.791	< 0.000	< 0.000
2SA	283,997.377	8305.509	930.176	7265.973	2241.213	69.931	247.504	31.400	1440.747	26.491	1139.207	92.480	14.356	3.123	11.099	5.201	0.411	0.814	0.718	< 0.000	< 0.000
3SA	304,528.153	7937.925	2933.593	7832.632	2553.923	< 0.000	274.792	44.525	1688.926	23.351	1303.945	90.439	16.350	5.443	13.344	7.198	1.133	1.082	0.592	< 0.000	< 0.000
4SA	279,690.674	67,110.116	4506.246	7395.764	3427.918	< 0.000	292.768	37.438	1967.667	53.882	1084.424	73.296	19.437	9.356	5.460	5.474	0.384	1.111	0.512	< 0.000	< 0.000
5SA	282,486.033	32,779.324	12,545.234	7420.534	3502.094	45.108	259.059	66.866	2406.671	31.619	832.350	791.425	36.197	32.053	6.640	5.587	1.936	1.418	0.488	< 0.000	< 0.000
6SA	312,326.000	31,525.885	2740.488	7916.181	1466.559	29.140	277.257	38.538	1688.999	37.315	779.122	93.219	18.773	8.488	10.213	5.968	0.395	1.212	0.459	< 0.000	< 0.000
7SI	280,548.827	3797.354	1382.829	7201.139	1417.497	36.746	270.151	31.869	1726.372	40.723	708.033	112.005	15.687	4.338	4.238	5.365	0.332	1.145	0.362	< 0.000	< 0.000
8SA	281,113.865	20,311.342	3481.251	7413.319	2584.777	105.322	251.766	43.841	1729.415	57.647	452.453	53.357	16.274	3.993	4.878	5.463	0.280	1.052	0.349	< 0.000	< 0.000
9SA	290,367.534	24,996.082	2449.835	7354.096	1024.023	15.467	251.908	32.898	1408.329	16.281	516.855	51.344	12.722	13.660	3.891	5.454	0.179	0.966	0.517	< 0.000	< 0.000
10SA	332,495.590	42,532.949	10,706.874	8200.448	865.454	44.167	286.603	39.680	1664.544	19.676	537.414	58.106	14.898	26.178	12.867	5.883	1.138	2.772	0.771	< 0.000	< 0.000
11SA	288,778.249	27,670.656	3069.011	7238.543	1032.485	5.224	251.647	36.239	1413.600	17.837	1100.854	43.896	14.656	5.133	17.609	5.176	0.227	0.862	0.520	< 0.000	< 0.000
12SA	297,473.396	24,417.074	3264.288	7871.002	3651.944	5.160	257.431	48.416	1797.784	16.720	392.671	60.346	19.009	20.753	13.006	5.918	0.042	0.932	0.405	< 0.000	< 0.000
13SA	293,107.971	12,617.820	1998.124	7390.599	1079.281	43.416	280.121	35.890	2077.120	19.241	347.108	51.425	11.415	10.105	9.031	6.534	0.442	1.165	0.257	0.049	< 0.000
14SA	307,038.356	11,511.362	4897.590	7674.265	1136.226	13.431	275.359	39.806	1723.864	24.483	289.667	51.977	14.530	13.224	5.754	5.771	0.223	1.161	0.315	0.047	< 0.000
15SA	315,511.719	67,492.456	4309.998	9629.993	1377.663	14.253	286.007	53.427	8066.864	18.750	447.968	173.691	23.222	16.843	4.119	7.454	31.776	80.747	0.247	0.213	0.024
16SI	312,446.385	4580.771	1789.174	7940.089	969.202	< 0.000	288.640	43.860	3000.433	18.023	438.338	175.244	15.824	3.631	0.253	5.763	0.345	1.095	0.400	0.099	0.018
1A	288,790.477	3347.612	4684.460	7440.853	1173.980	10.400	256.033	43.459	1749.125	19.140	295.005	162.992	18.950	4.030	0.242	6.653	0.350	1.339	0.144	0.101	< 0.000
1C	285,997.818	12,406.925	1697.972	7334.726	1747.675	< 0.000	249.934	39.484	2115.489	20.291	317.436	199.634	19.990	7.286	6.315	6.626	0.292	0.905	0.332	0.000	< 0.000
1H	288,856.831	9199.170	2879.171	7586.830	1402.966	5.964	294.388	39.565	1847.240	16.707	448.209	165.817	15.027	8.629	10.878	5.786	0.409	1.601	0.272	0.049	< 0.000
1 M	290,762.225	37,954.857	5237.920	7529.357	2288.529	< 0.000	264.986	41.877	1775.445	18.223	355.037	165.788	19.987	20.811	7.084	5.206	0.404	1.477	0.349	0.000	< 0.000
1N	292,509.280	52,745.399	3908.204	7591.719	1402.945	< 0.000	265.116	39.810	1665.172	22.791	354.969	170.212	18.666	26.782	4.608	6.815	4.001	4.630	0.124	0.046	< 0.000
2N	284,041.444	56,812.616	5522.720	7959.938	1291.200	20.265	253.962	51.488	1633.806	23.482	304.856	289.900	19.457	23.877	9.171	6.731	0.345	1.116	0.306	0.145	< 0.000
3N	303,899.893	35,361.618	4415.262	7686.440	1778.768	< 0.000	296.997	46.033	1791.849	25.613	339.701	40.839	17.342	15.874	15.029	6.884	0.580	1.107	0.240	0.098	< 0.000
1O	293,661.951	13,734.505	2882.848	7386.329	3214.814	49.760	269.926	44.927	1886.242	18.779	331.785	44.096	19.139	4.873	3.350	6.250	0.415	1.007	0.069	0.052	< 0.000
2O	314,677.233	10,953.003	1828.682	8112.012	1746.993	< 0.000	285.354	48.039	2208.419	20.003	298.959	63.230	18.530	6.823	6.798	6.850	1.227	1.444	0.375	0.000	< 0.000
1 T	300,517.595	31,674.256	3448.278	7414.057	1337.765	5.981	263.058	101.552	18,879.005	60.590	318.514	56.743	17.481	7.094	8.147	6.460	0.269	0.947	0.210	0.099	< 0.000
1Z	292,569.920	22,992.095	3047.725	7161.934	1525.829	< 0.000	265.416	51.879	6518.432	16.988	211.163	50.634	14.822	14.790	3.310	7.121	0.186	0.883	0.296	0.048	< 0.000

*Abbreviations*: 1N, *Nepeta cilicia*; 2N, *Nepeta isaurica*; 1 T, *Thymus cilicicus*; 1O, *Origanum leptocladum*; 1Z, *Ziziphora clinopodioides*; 1SI, *Sideritis hololeuca*; 2SA, *Salvia tomentosa*; 3SA, *Salvia pisidica*; 1C, *Cyclotrichium origanifolium*; 4SA, *Salvia heldreichiana*; 5SA, *Salvia absconditiflora*; 6SA, *Salvia quezelii*; 7SI, *Sideritis libanotica* subsp. *violascens*; 3N, *Nepeta sulphuriflora*; 8SA, *Salvia caespitosa*; 9SA, *Salvia dichroantha*; 1A, *Ajuga chamaepitys* subsp. *chia*; 10SA, *Salvia viridis*; 1 M, *Marrubium globosum* subsp. *globosum*; 11SA, *Salvia aucheri* subsp. *canescens*; 12SA, *Salvia blepharochlaena*; 13SA, *Salvia candidissima* subsp. *occidentalis*; 2O, *Origanum vulgare*; 14SA, *Salvia albimaculata*; 15SA, *Salvia hypargeia*; 1H, *Hyssopus officinalis* subsp. *officinalis*; 16SI, *Sideritis argyrea*

The definition of "trace element" as outlined in the IUPAC Compendium of Chemical Terminology, second edition, sets a threshold: "Any element having an average concentration of less than about 100 parts per million atoms or less than 100 μg/g." However, advancements in analytical techniques have pushed detection capabilities further. In many fields, the upper boundary of the "trace" definition now falls short of the analytical precision achievable today. Consequently, terms like "ultra-trace analysis" have emerged to delineate this domain. Although there is no universally agreed-upon range for ultra-trace analysis, it generally refers to elements with mass fractions below 10 − 6 and 10 − 8 g/g (1 ppm and 10 ppb), respectively [21]

## Discussion

### Qualitative Analysis of Secondary and Primary Metabolites

A comprehensive review of literature from 2002 to 2018 revealed various classes of secondary metabolites in this family, including flavonoids, fatty derivatives, and sterols [13].

In a review article, a total of 217 articles were selected from the initial search focusing on *Lamiaceae*, specifically those recognizing Mexican genera and species. The bioactive constituents identified in these genera predominantly include terpenes (both volatile and non-volatile) and phenolic compounds, particularly flavonoids in the form of glycosides and aglycones [22].

A review provided an overview of *Nepeta* species, focusing on their phytochemical characteristics. Terpenoids and phenolic compounds were predominantly identified through the application of chromatographic and spectroscopic techniques [23]. In another review, major constituents as nepetalactones, iridoids and their glucosides, diterpenes, triterpenes, and flavonoids, as well as essential oil, have been identified within *Nepeta* species [24]. The phytochemical composition and trace elements of *Nepeta suavis* were examined in an analysis. The findings unveiled the existence of bioactive components, including flavonoids, alkaloids, phenols, saponins, and tannins. Furthermore, the herb proved to be a rich reservoir of essential minerals such as Na, K, Ca, Mg, Zn, Fe, and P [25]. The volatile oils stand out as the primary constituents of the *Thymus* genus. In addition to these, the genus is rich in flavonoids, phenylpropanoids, tannins, organic acids, terpenoids, and phytosterols [26]. The *Origanum* genus exhibits chemical variations. Oregano, recognized for its distinctive flavor, is primarily associated with several plant species known for producing essential oils rich in carvacrol. Additionally, the genus consists of a diverse range of compounds, including terpenes, phenols such as phenolic acids, and flavonoids [27]. Numerous chemical constituents have been elucidated within the *Sideritis* genus, encompassing terpenes, flavonoids, essential oils, iridoids, coumarins, lignans, and sterols. Diterpenes, flavonoids, and essential oils are consistently present in nearly every species, serving as the primary components responsible for the pharmacological activity [28].

In a study, the aerial parts of *Sideritis* and *Origanum* species were analyzed for mineral contents, flavonoids, total phenols, and anthocyanins. K was found to be high in both plant species. In *Sideritis*, K contents ranged from 10,184.91 mg/kg (*Sideritis libanotica* subsp. *linearis*) to 17,182.86 mg/kg (*S. hispida*), while in *Origanum*, they ranged from 10,265.40 mg/kg (*Origanum majorana*) to 21,293.79 mg/kg (*O. vulgare*). Crude protein contents in *Sideritis* varied between 1.55% (*S. libanotica* subsp. *linearis*) and 7.83% (*S. perfoliata*), whereas protein contents in *Origanum* species ranged from 1.99% (*O. leptocladum*) to 5.51% (*O. vulgare*). Flavonoid contents in *Sideritis* plants ranged from 246.34 (*S. libatotica* subsp. *linearis*) to 2013.33 (*S. hololeuca*), and in *Origanum* plants, they varied from 345.38 (*O. onites*) to 1730.47 (*O. majorana*). For *Origanum*, K contents ranged between 10,265.40 mg/kg (*O. majorana*) and 21,293.79 mg/kg (*O. vulgare*) [29].

The terpenoids and flavonoids constitute the primary secondary metabolite constituents of *Salvia*, with over 80% being terpenoids, particularly abietane and clerodane diterpenoids. Notably, sesquiterpenoids and triterpenoids are relatively scarce in *Salvia* species. Numerous studies highlight the presence of flavonoids, triterpenoids, and monoterpenes, particularly in the flowers and leaves, while diterpenoids are predominantly found in the roots. However, literature surveys indicate that certain American *Salvia* species also contain diterpenoids in aerial parts, and in some *Salvia* species, triterpenoids and flavones are present in the roots [30]

In a research, *Salvia officinalis*, *S. sclarea*, *S. pratensis*, and *S. nemorosa* originating from Hungary and Transylvania were investigated for their tannin and flavonoid content. Significant differences (p < 0.05) were observed in tannin content between Hungarian and Transylvanian *S. officinalis* and flavonoid content between Hungarian and Transylvanian *S. sclarea*. Chromium content was notably high in all examined species. The element concentrations also differed significantly in aqueous extracts, with distinct dissolution rates. Notably, Hungarian *S. officinalis* exhibited elevated concentrations of Al, Fe, Mn, and Ti, possibly linked to soil pollution. Zinc accumulation was highest in Transylvanian *S. officinalis* and *S. pratensis*, while Hungarian *S. nemorosa* demonstrated elevated Li content. Chromium content was notably high in Hungarian *S. officinalis* and *S. sclarea*. Dissolution rates of elements varied widely among sage teas, showcasing significant differences in element concentrations (Al, B, Ba, Cu, Fe, K, Mg, Mn, Na, P, S, Zn) and dissolution characteristics based on the sample and the element studied [31].

The genus *Ziziphora* has been extensively studied, and previous reports in the literature highlight its species as rich sources of valuable bioactive compounds, including sterols, fatty acids, caffeoyl derivatives, and flavonoids. Notably, among the different groups of natural compounds, flavonoids, flavones, and their derivatives exhibit the highest frequency in the separated and characterized bioactive compounds, contributing significantly to each profile [32].

In literature, studies have indicated that *Cyclotrichium* essential oils are abundant in phenolic and alcoholic compounds, along with monoterpenes. Phenolic compounds are widely believed to be abundant in most *Cyclotrichium* species. *C. origanifolium* is rich in flavonoid [33]. According to our knowledge, this is the first elemental analysis conducted on the *Cyclotrichium* genus.

A comprehensive review aimed to provide an in-depth summary of the genus *Ajuga* L. Currently, more than 280 chemical constituents have been isolated and characterized from *Ajuga* species, with neo-clerodane diterpenes and diterpenoids, phytoecdysteroids, flavonoids, and iridoids identified as the major bioactive compounds [34]. This study represents the inaugural investigation into the elemental analysis of *Ajuga chamaepitys*.

In a comprehensive literature review on the genus *Marrubium*, chemical characteristics were investigated. The biological effects of the plants are often attributed to the presence of diterpenes, sterols, phenylpropanoids, and flavonoids [35].

Hyssop (*Hyssopus sp.*) genus has been known with volatile oils. The genus also containe flavonoid in leaves, flowers, stalks, and roots during full flowering. Furthermore, hyssop seeds have fatty acid content in oil [36].

### Elemental Analysis

In a study conducted similarly to our research, the elemental composition of 45 medicinal plant species belonging to the *Lamiaceae* family in the Republic of Moldova was investigated. Various elements, including essential, rare earth, and trace elements (measured in μg/L), as well as major elements such as Ca, Cl, K, Mg, and Na (measured in mg/L), were determined in the analyzed samples. Despite the presence of the toxic element As in all plants, its concentration in the majority of samples was below the limit set [37].

The study conducted in Pakistan, aimed to investigate the phytochemical, and elemental aspects of five crucial medicinal plants from the *Lamiaceae* family, namely *Mentha longifolia*, *M. piperita*, *M. spicata*, *Ocimum basilicum*, and *Rosmarinus officinalis*, collected in Peshawar district. Quantitative analysis of macro- and microminerals identified 13 elements (C, N, O, Mg, K, P, S, Ca, Al, Si, Fe, Cl, and Na) present in varying amounts among species. Methanol extracts from leaves were analyzed for phytochemical constituents such as saponins, flavonoids, tannins, terpenoids, phlobatannins, steroids, and anthraquinones [38].

Another research aimed to analyze the elemental content of six medicinal plant species from the *Lamiaceae* family, predominantly found in the western part of Romania. The findings indicate that these medicinal plants are rich sources of nutrients, with high potassium (K) content, followed by calcium (Ca), magnesium (Mg), iron (Fe), and zinc (Zn) [39].

A study explored the mineral elements of extract from *N. italica* subsp. *cadmea*. Mineral elements (P, Mg, K, Fe, Cu) were found in the extract [40]. A paper focused on the analysis of trace elements in the Indian medicinal plant *Nepeta hindostana*. The plant was identified as a rich source of essential trace elements as Na, K, Mg, Zn, Fe, and Mn [41].

A study investigated the concentrations of eleven mineral and trace elements (Mg, Ca, K, Fe, Mn, Al, Zn, Cu, Cd, Ni, and Pb) in various *Thymus* species in Turkey, including the *Thymus cilicicus*. The elemental concentrations (mg/g) in *T. cilicicus* were found to be 0.40 ± 0.02 (Pb), 0.20 ± 0.10 (Ni), 0.04 ± 0.01 (Cd), 10.70 ± 2.30 (Cu), 40.80 ± 4.70 (Zn), 191.0 ± 8.0 (Al), 19.0 ± 2.0 (Mn), 189.0 ± 2.0 (Fe), 12,250.0 ± 672.0 (K), 14,417.0 ± 1909.0 (Ca), and 4127.0 ± 1200.0 (Mg). *T. cilicicus* stands out as particularly rich in essential elements, with the highest concentrations observed for Ca, K, and Mg [42].

In a study, elemental concentrations in *Ziziphora* medicinal plants were analyzed. Samples were collected from the Eynali mountain region in the north of Tabriz, Iran. The study revealed varying concentrations of Al, Ca, K, Mg, Mn, Na, V, Cl, and Ti elements in powdered *Ziziphora* plants. Notably, *Ziziphora* plants exhibited richness in essential elements such as Mg, K, and Ca, all of importance for human health. Importantly, the concentration of the potentially toxic element, Al, was determined to be below the levels permitted by the World Health Organization (WHO) [43].

In research, to rationalize its medicinal applications and establish a biogeochemical link, the mineral elements (Na, K, Ca, Mg, Zn, Mn, Cu, Fe, Cr) present in the leaves and roots of *Ajuga bracteosa* were investigated. The herb exhibited comparatively higher amounts of chromium (25 mg per 100 g in leaves and 20 mg in roots), which may be associated with its traditional use as a remedy for diabetes. Additionally, the herb contains significantly higher levels of potassium (139 mg per 100 g in leaves and 159 mg in roots) compared to sodium (21 mg per 100 g in leaves and 29 mg in roots), suggesting a potential correlation with its use in managing hypertension [44].

According to our knowledge, this is the first elemental analysis conducted on the *Marrubium globosum.* In another study, instrumental neutron activation analysis identified twenty-two chemical elements in *Marrubium vulgare*. The study revealed that K was the dominant chemical element in the plant, constituting 4.40% of the mass. Ca and Fe mass fractions were also relatively high. Importantly, potential toxic elements in this *Lamiaceae* plant were found to be within the safety limits recommended by WHO/FAO [45].

A study considered four harvest heights (15, 25, 35, and 45 cm from the tip of the *Hyssopus officinalis*) along with the residual stalks. Dependent variables included the accumulated content of elements (N, K, P, Ca, Mg, Cu, Zn, and Pb) at different heights. Results revealed that moving from the upper shoots towards the ground led to an increase in Mg, Ca, Cu, Zn, and Pb content by 22.67%, 43.74%, 12.87%, 39.02%, and 85.04%, respectively. Conversely, a downward trend was observed for N (50.16%) and K (6.41%) content, while an upward trend was noted for P (29.06%) content. In residual stalks, moving from upper shoots to the ground resulted in decreased Mg, Ca, Cu, Zn, and Pb contents by 1.01%, 21.03%, 9.11%, 17.02%, and 51.06%, respectively. However, N and P contents increased by 60.59% and 3.15%, respectively, with a 34.74% increase in P content [46].

The comprehensive analysis of primary and secondary metabolites, alongside elemental composition evaluation of methanol extracts, offers profound insights into the characteristics and potential functionalities of the samples. The ubiquitous presence of flavonoids and tannins across all samples hints at the antioxidant capabilities and potentially broader health-promoting effects of the plants under study. Flavonoids, renowned for their antioxidant and anti-inflammatory properties, stand as focal points in numerous health studies. Conversely, tannins, recognized for antimicrobial and antiviral attributes, contribute to the overall pharmacological profile of these plants. The absence of cardioactive heterosides suggests a lack of pronounced effects on cardiac function, which could be advantageous or constraining depending on the intended application of these botanicals. The detection of coumarin in varied samples sparks interest due to its diverse pharmacological spectrum, encompassing anticoagulant, anti-inflammatory, and antimicrobial facets. This discovery implies that the plants may harbor therapeutic potential extending beyond mere antioxidant capacities. The presence of lipids in several samples and the occurrence of starch in select ones accentuate the nutritional diversity inherent in these botanicals. Lipids, vital constituents of cellular membranes, and starch, a pivotal energy source, contribute to the overall nutritional value of these plants. Regarding elemental composition, the elevated levels of sodium and potassium throughout the samples hint at their potential mineral richness. However, the identification of heavy metals like aluminum, chromium, and arsenic raises concerns regarding potential toxicity, particularly in samples exhibiting higher concentrations of these elements. The provided text offers a comprehensive overview of studies focusing on the Lamiaceae family, exploring the elemental composition and phytochemical characteristics of various plant species within this family. These studies shed light on the diverse array of bioactive compounds present in Lamiaceae plants, ranging from essential minerals to secondary metabolites like flavonoids, terpenoids, and phenolic compounds. One notable aspect highlighted in the text is the prevalence of essential elements such as K, Ca, Mg, and Fe across different Lamiaceae species. These elements play crucial roles in various physiological processes, including enzymatic reactions, cellular signalling, and structural support. The abundance of these minerals underscores the potential nutritional significance of Lamiaceae plants in human diets. Additionally, the presence of bioactive compounds like flavonoids, terpenoids, and phenolic compounds underscores the therapeutic potential of Lamiaceae plants. These compounds have been associated with various pharmacological activities, including antioxidant, anti-inflammatory, and antimicrobial properties. The diverse chemical profile of Lamiaceae plants suggests their potential utility in traditional medicine and as sources of novel therapeutic agents. Furthermore, the studies discussed in the text also address the elemental variability among different Lamiaceae species, as well as the influence of environmental factors on elemental composition. Understanding these variations is crucial for assessing the nutritional quality and potential health benefits of Lamiaceae plants, as well as for elucidating their ecological roles and adaptation strategies. Overall, the findings presented in the text underscore the importance of interdisciplinary approaches in studying plant chemistry and ecology. Integrating elemental analysis with phytochemical profiling provides valuable insights into the nutritional, medicinal, and ecological significance of Lamiaceae plants, ultimately contributing to our understanding of their roles in human health and natural ecosystems.

In summary, these findings underscore the intricate nature of plant metabolites and emphasize the necessity for exhaustive analysis in deciphering their potential health implications. Further investigations, encompassing pharmacological and toxicological inquiries, are imperative for a comprehensive understanding of the therapeutic prospects and safety profiles associated with these botanicals.

## Conclusion

In conclusion, the comprehensive analysis of 27 medicinal plant species from the *Lamiaceae* family in Turkey revealed a diverse spectrum of constituents, encompassing both organic and inorganic elements. The elemental composition, including 18 endemic species, was determined using ICP/MS, highlighting the presence of essential, rare earth, and trace elements at mg/kg concentrations. Major elements such as Na and K were found in elevated levels, demonstrating the richness of these plants in important nutrients. Despite the widespread detection of the toxic element As (arsenic), the concentrations in most samples remained below the World Health Organization's established threshold, assuring the safety of these medicinal plants. The inclusion of specific primary and secondary metabolites, such as flavonoids, tannins, saponins, coumarin, starch, lipids, and carbohydrates, further underscored the diverse chemical composition of these plants. Overall, this study provides valuable insights into the elemental and chemical profiles of medicinal plants from the *Lamiaceae* family, contributing to our understanding of their potential therapeutic properties and nutritional benefits. The presence of essential elements in trace amounts, coupled with the relatively low levels of toxic elements, reinforces the potential of these plants as valuable resources for traditional and modern medicine.

## Data Availability

Data will be made available on request.
